# Supplementary material for: The coincidence of ecological opportunity with hybridization explains rapid adaptive radiation in Lake Mweru cichlid fishes
Source: Nat Commun. 2019 Dec 3;10:5391. doi: 10.1038/s41467-019-13278-z (PMC6890737; doi:10.1038/s41467-019-13278-z)
Supplement: Supplementary file 1 — Supplementary Information [file 41467_2019_13278_MOESM1_ESM.pdf]

Supplementary Information

The coincidence of ecological opportunity with hybridization explains rapid adaptive radiation in Lake Mweru cichlids

Meier et al.

Supplementary Figures and Tables

Supplementary Figure 1: Lake depth does not explain the lack of speciation in Lake Bangweulu and the presence of multiple adaptive radiations in Lake Mweru
Supplementary Figure 2: The interacting radiations of Lake Mweru.
Supplementary Figure 3: Comparison of nuclear (left) and mitochondrial trees (right) reveal considerable evidence for monophyly, yet rampant cytonuclear discordance.
Supplementary Figure 4: Morphological and genomic PCA of radiations in Lake Mweru.
Supplementary Figure 5: Size distribution of the different clades in Lake Mweru.
Supplementary Figure 6: Dated mitochondrial phylogeny suggests a recent and broadly overlapping age of the four radiations in Lake Mweru.
Supplementary Figure 7: Phylogenetic tree and ADMIXTURE analysis of the serranochromine samples.
Supplementary Figure 8: fineRADstructure coancestry matrix of “orthochromines” reveals absence of recent gene flow between *Pseudocrenilabrus* and *Orthochromis* species and supports homogenous genomic compositions of the *Pseudocrenilabrus* radiation.
Supplementary Figure 9: fineRADstructure coancestry matrix of serranochromines reveals homogenous patterns of haplotype sharing within each radiation and except for one putative case of backcrossing, shows no evidence for recent gene flow.
Supplementary Table 1: Mitochondrial chronogram calibration sets and age estimates.

Supplementary Notes

Supplementary Note 1: Background on Lakes Mweru and Bangweulu
Supplementary Note 2: Taxonomic diversity
Supplementary Note 3: Species diversity in the Lake Mweru radiations *Pseudocrenilabrus* radiation
Supplementary Note 4: Comparison of Lake Mweru haplochromines to those of Lake Victoria
Supplementary Note 5: Evidence for hybrid origins of the adaptive radiations in Lake Mweru

Supplementary References

Supplementary Figures and Tables

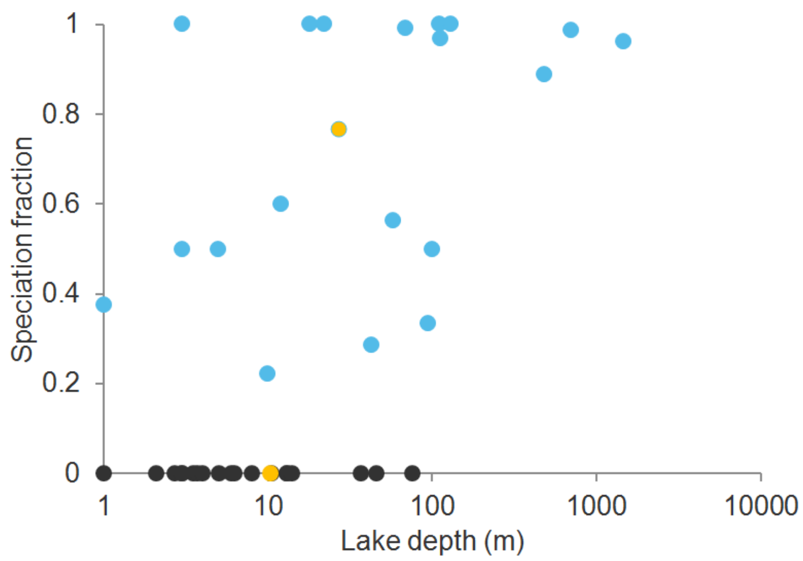

Supplementary Fig. 1: Lake depth does not explain the lack of speciation in Lake Bangweulu and the presence of multiple adaptive radiations in Lake Mweru. Blue and black dots depict lakes with and without adaptive radiation, respectively, whereas orange dots highlight Lakes Mweru and Bangweulu. The fraction of species that evolved through *in situ* speciation (speciation fraction) is not significantly correlated with lake depth. Lakes similar in depth as Lakes Bangweulu and Mweru can host cichlid communities that are fully assembled through colonization or completely derived from *in situ* speciation, or anything in between. Figure adapted from Wagner et al.[1].

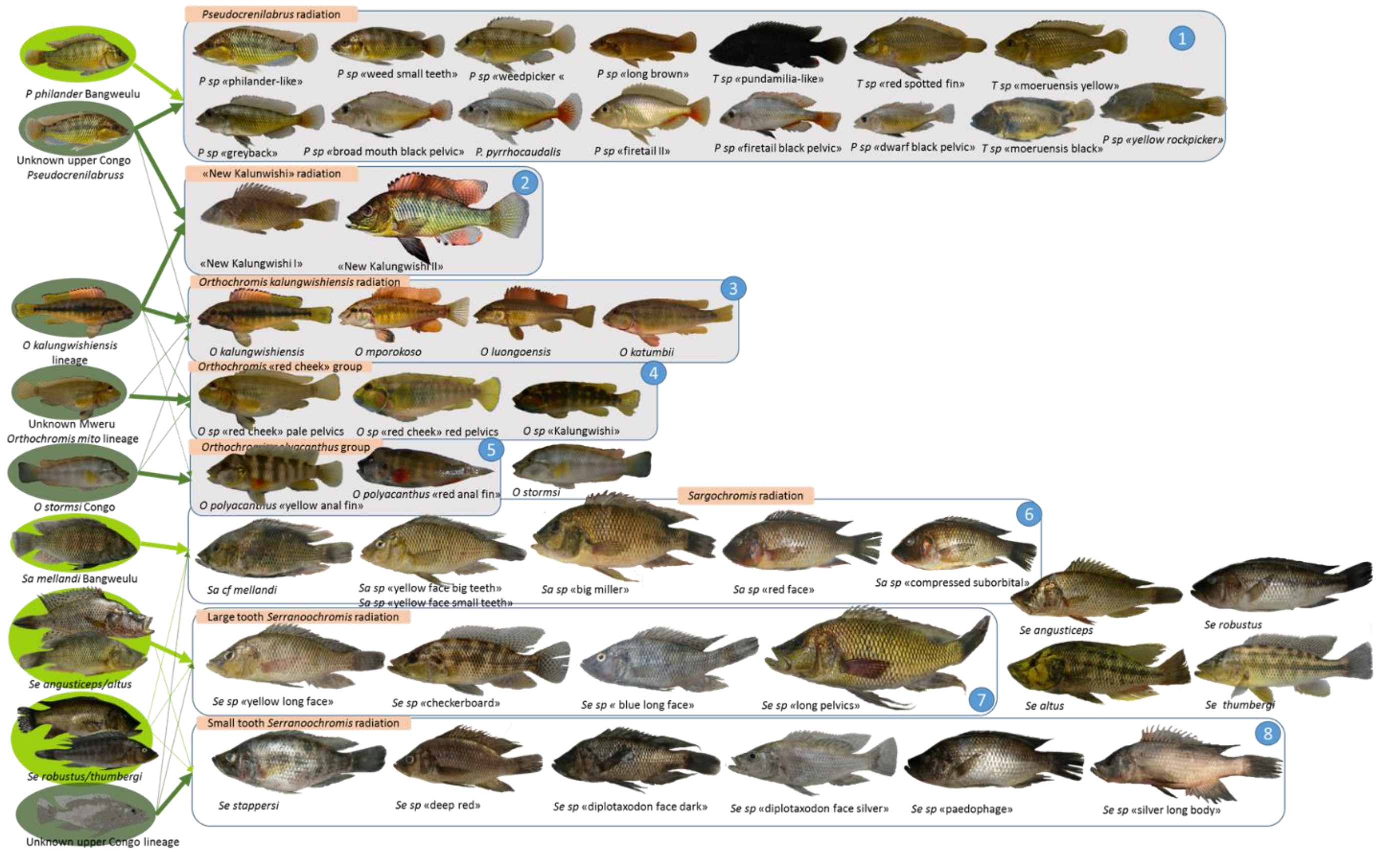

Supplementary Figure 2: The interacting radiations of Lake Mweru. Representative photos of the species of all the radiations in Lake Mweru, their putative ancestral lineages (C, Z refer to lineages of Congolese and Zambezi origins), and the four non-endemic haplochromines of Lake Mweru. All nine ancestral lineages have contributed genetic material to endemic radiations, except perhaps *Orthochromis stormsi* if the *O. polyacanthus* clade is not considered a radiation. Radiations are boxed in a thin line. Arrows indicate ancestry of radiations pointing from the ancestor to the radiations (dashed arrow indicates uncertainty about the direction of gene flow). Most radiations received genes from more than one colonizing lineage. Thickness of arrows denotes major (thick) and minor (thin) contribution to admixture. The *Orthochromis*/*Pseudocrenilabrus* radiations at the top (grey boxes) and the serranochromine radiations at the bottom (white boxes) are distantly related and did not exchange genes with each other. Radiations 1, 5, 6, 7, 8 are entirely lacustrine. Radiations 2 and 3 are confined to inflowing rivers, and radiation 4 has representatives in the lake (*O*. sp. “red cheek”) and in the rivers (*O*. sp. “Kalungwishi”). The “New Kalungwishi I” individual was photographed by Numel Phiri and Cyprian Katongo, the “New Kalungwishi II” (from Luongo River) and the four individuals of the *O. kalungwishiensis* radiation were photographed by Hans van Heusden, and *O. stormsi* by Ulrich Schliewen. All other photos were taken by Ole Seehausen.

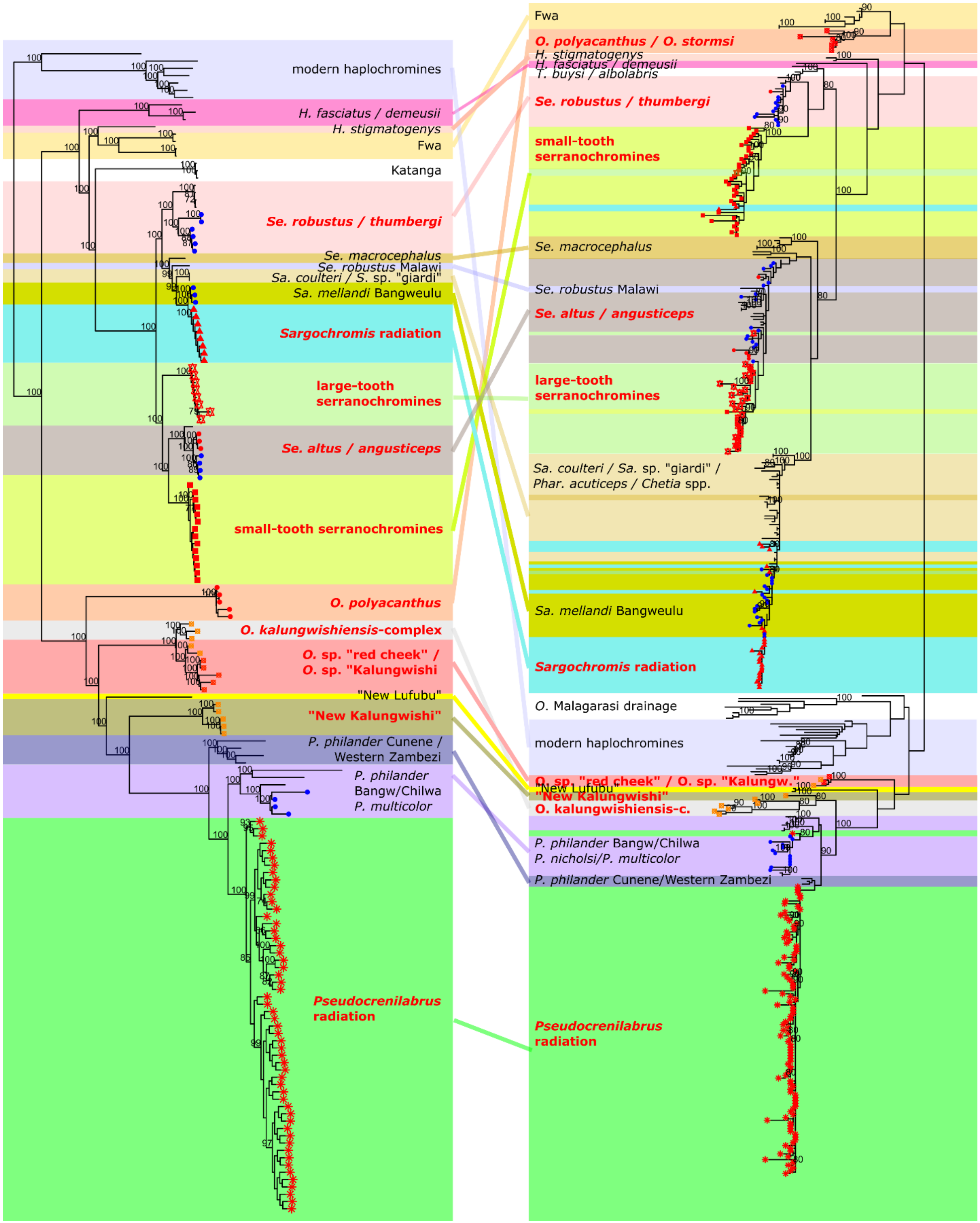

Supplementary Figure 3: Comparison of nuclear (left) and mitochondrial trees (right) reveal considerable evidence for monophyly, yet rampant cytonuclear discordance. Cichlids in Lake Mweru, Bangweulu or rivers of the Lake Mweru drainage system are highlighted with red, blue or orange symbols, respectively. Red bold labels indicate occurrence of the group in Lake Mweru or its major inflowing rivers. For a version with individual labels, see Supplementary Data 1.

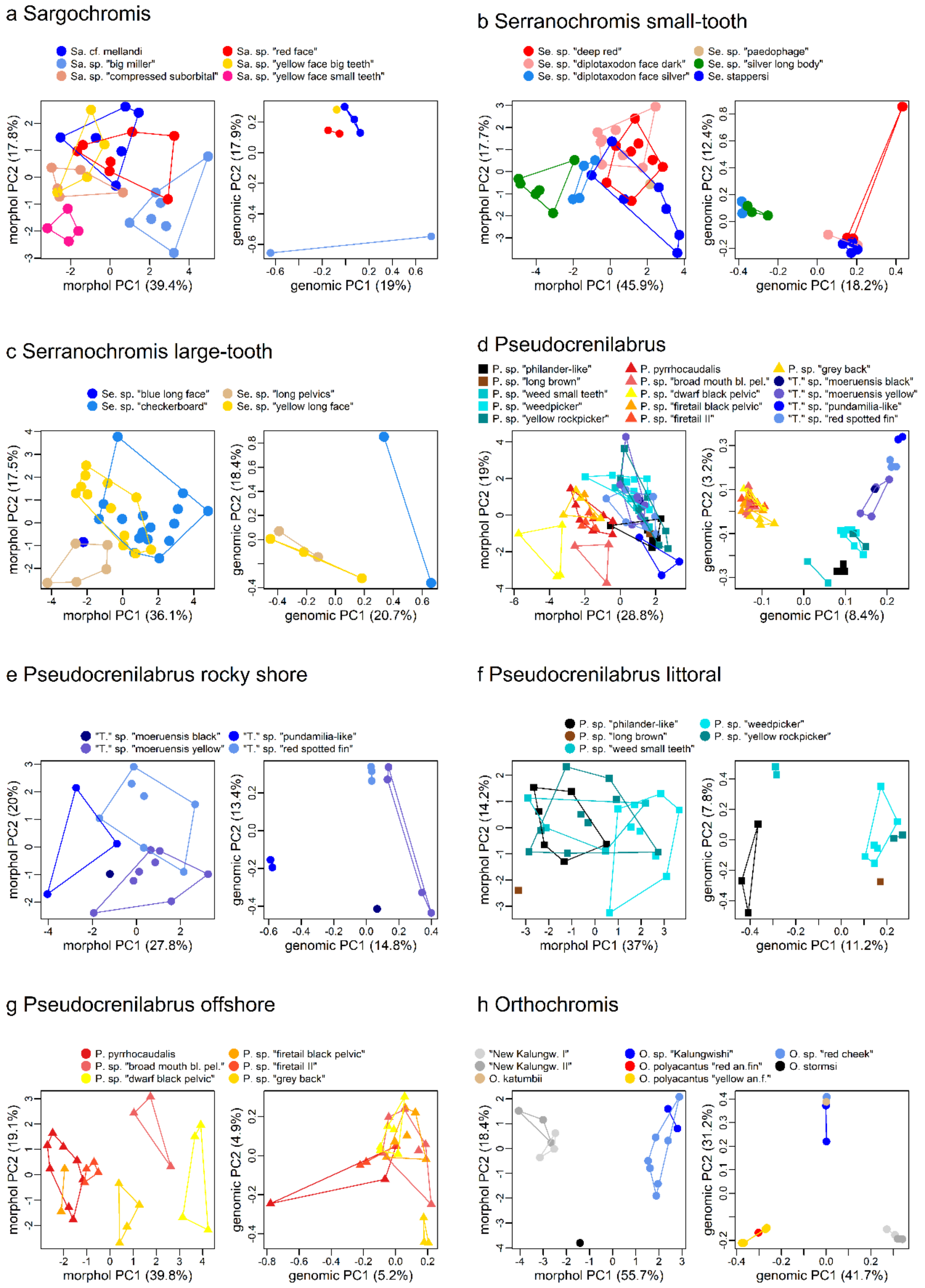

Supplementary Figure 4: Morphological and genomic PCA of radiations in Lake Mweru. The habitat group of the Pseudocrenilabrus radiation are shown separately. The underlying data and R script are given on Zenodo doi:10.5281/zenodo.3435419.

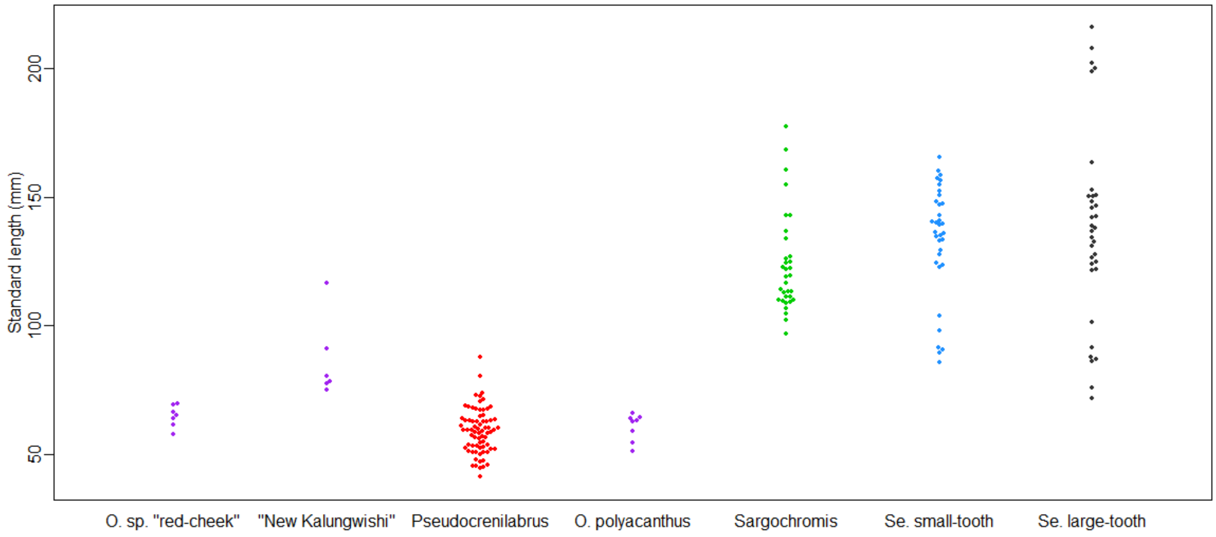

Supplementary Figure 5: Size distribution of the different clades in Lake Mweru. The underlying data and R script are provided on Zenodo doi:10.5281/zenodo.3435419.

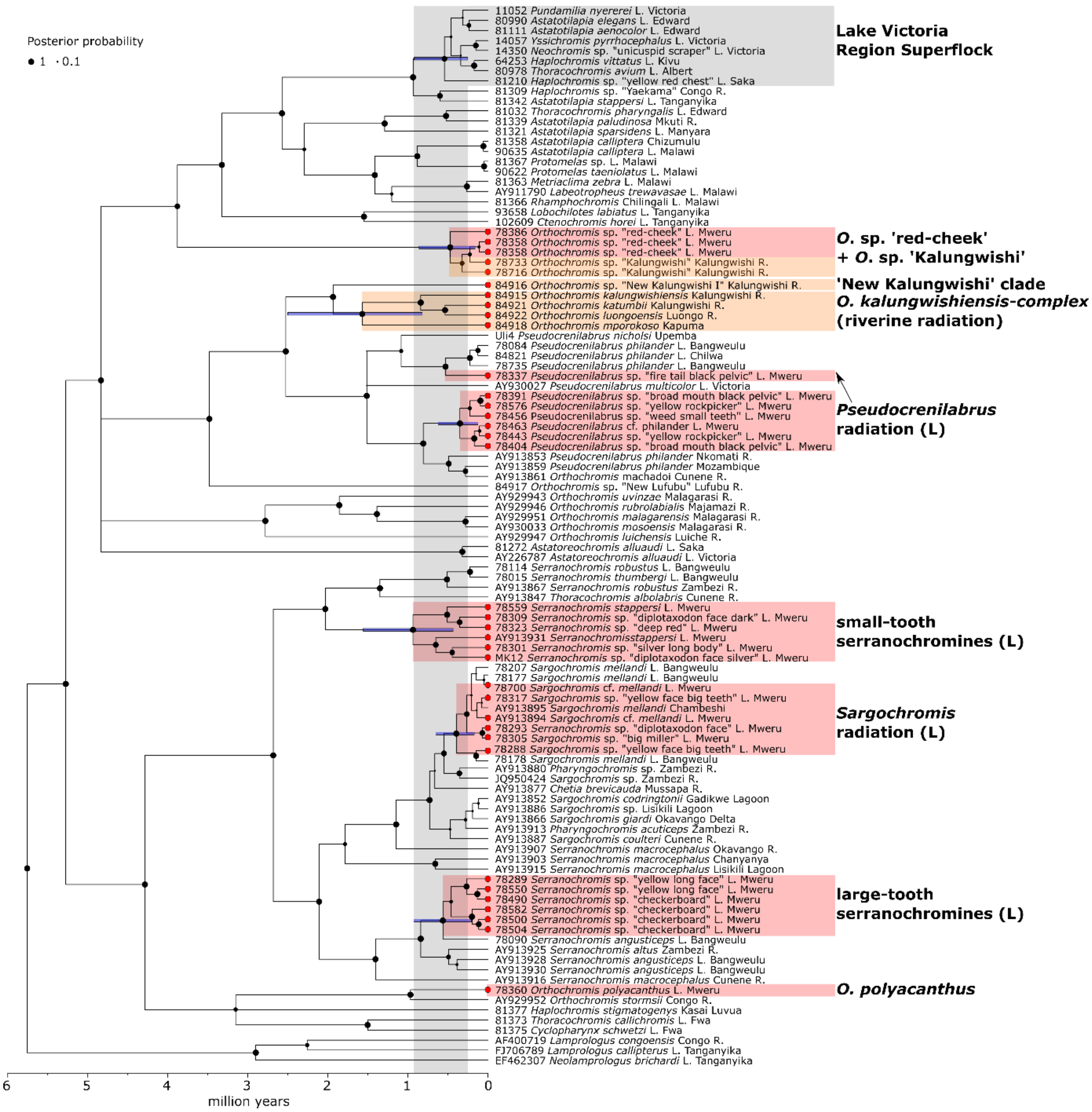

Supplementary Figure 6: Dated mitochondrial phylogeny suggests a recent and broadly overlapping age of the four radiations in Lake Mweru. The four lacustrine radiations (indicated with an L) show broad overlap of confidence intervals around their age estimates with the origin of the Lake Victoria Region Superflock of cichlids (grey, estimated to 150,000 years). Taxa occurring in Lake Mweru or adjacent rivers are highlighted with red or orange background coloration, respectively. The confidence intervals (CI) around the lacustrine radiation age means range from 0.12 Mya (lower bound of the CI of the *Pseudocrenilabrus* radiation) to 1.64 Mya (upper bound of the CI of the *Serranochromis* small-tooth radiation) using the non-cichlid fossil calibration set shown here. Values for all calibration sets are given in Supplementary Table 1 and all BEAST trees are provided on Zenodo

doi:10.5281/zenodo.3435419. Note that the age of each radiation is inferred as the age of the node subtending the vast majority of the mitochondrial haplotypes of each respective radiation, excluding individual with cytonuclear mismatch. These clades likely reflect the onset of population growth and adaptive radiation following the colonization of Lake Mweru. The single *Pseudocrenilabrus* individual with a mitochondrial haplotype that is more closely related to *Pseudocrenilabrus* from Lake Bangweulu and Lake Chilwa than to the majority Mweru clade (indicated with an arrow) likely represents the Zambezian ancestral lineage of *Pseudocrenilabrus*. The estimated age of the radiations coincides with the Luapula-Chambeshi river capture event which is estimated to about 1 million years ago.

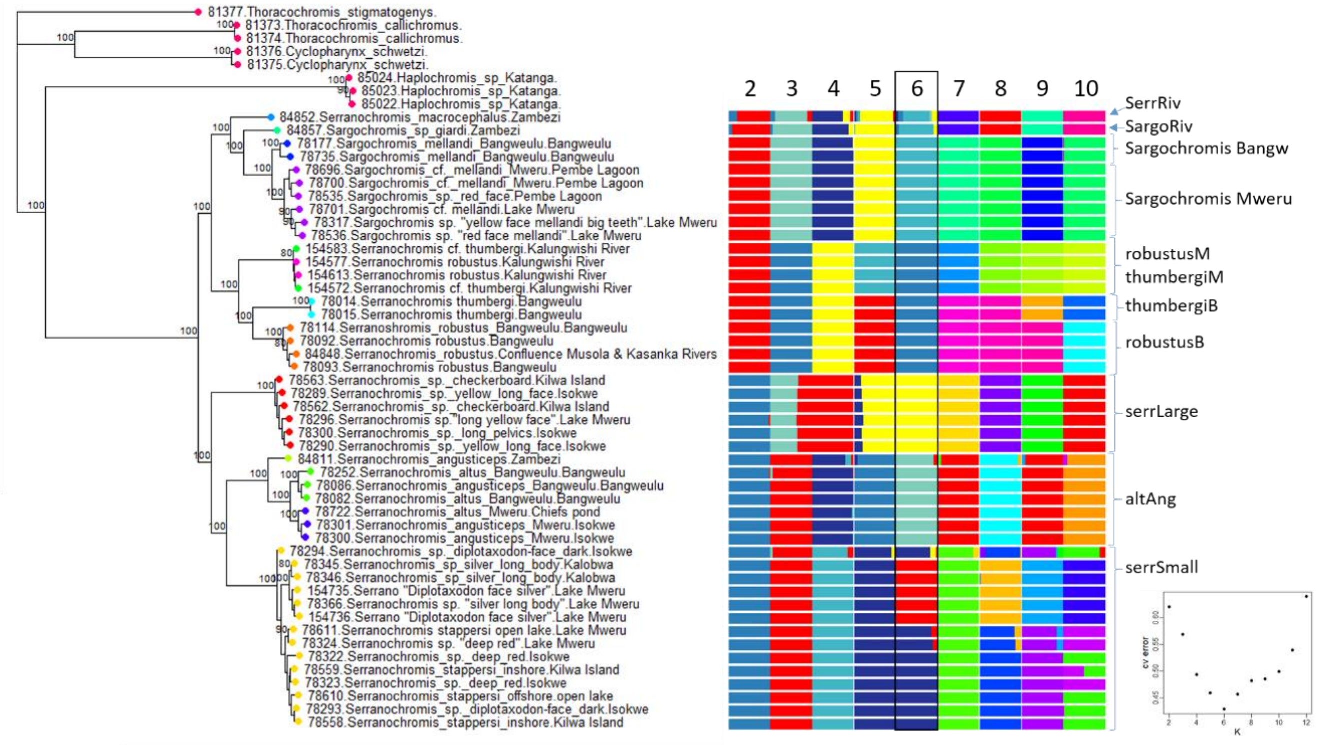

Supplementary Figure 7: Phylogenetic tree and ADMIXTURE analysis of the serranochromine samples. The inlet shows the cv errors for different number of clusters (K) indicating that six clusters is optimal.

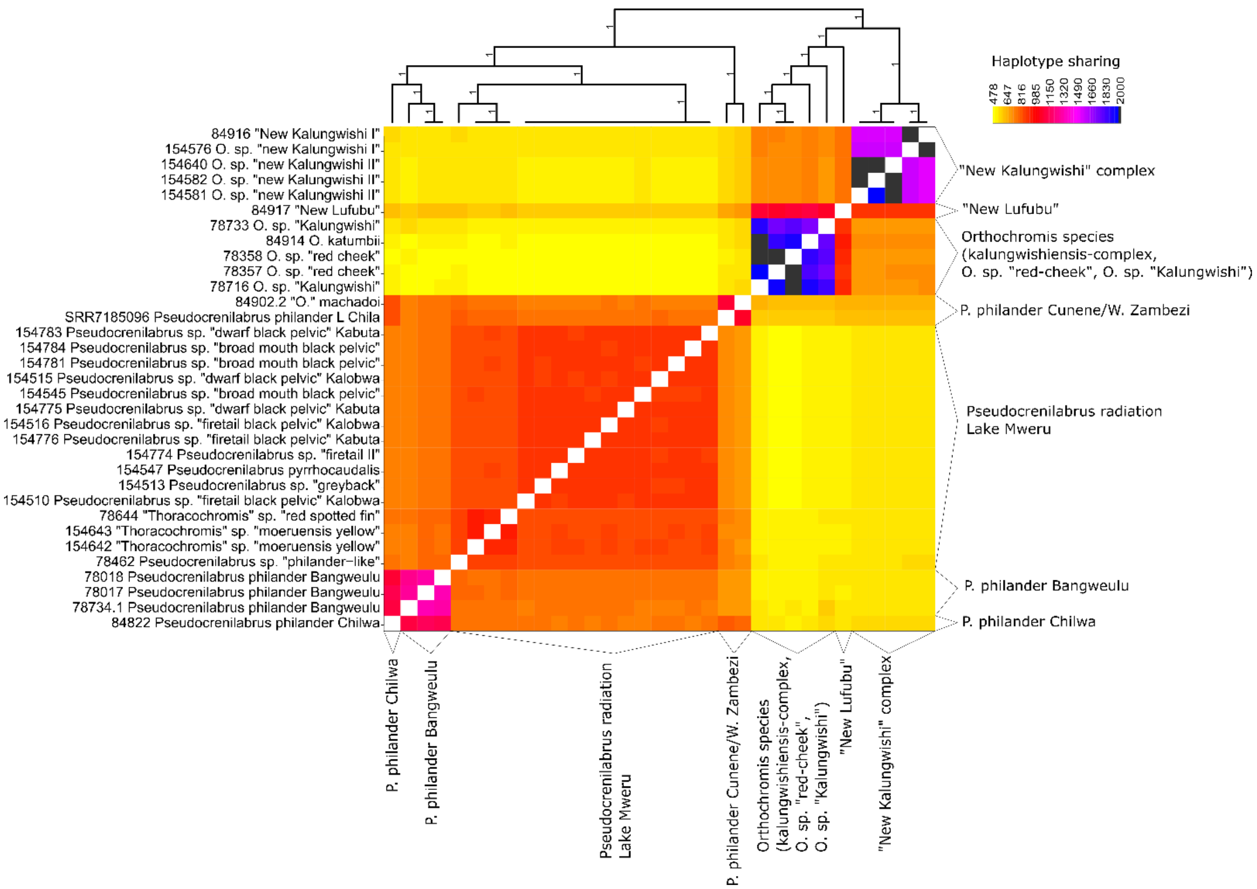

Supplementary Figure 8: fineRADstructure coancestry matrix of "orthochromines" reveals absence of recent gene flow between *Pseudocrenilabrus* and *Orthochromis* species and supports homogenous genomic compositions of the *Pseudocrenilabrus* radiation. We used fineRADpainter to infer the closest relative (donor) for each haplotype (two per individual per RADlocus), and then ran fineRADstructure to infer the coancestry matrix. Donor individuals are shown in columns and recipient individuals are shown in rows. Despite clustering with the "New Kalungwishi" species complex, the "New Lufubu cichlid" shows high haplotype sharing with all *Orthochromis* species and all *Pseudocrenilabrus* species indicating that it may be a recent mix of these two genera. On the other hand, the "New Lufubu cichlid" is only a single individual and any potential donors of haplotypes must thus be a rather distant relative. The fineRADstructure output files and the R script used to produce this figure is provided on Zenodo doi:10.5281/zenodo.3435419.

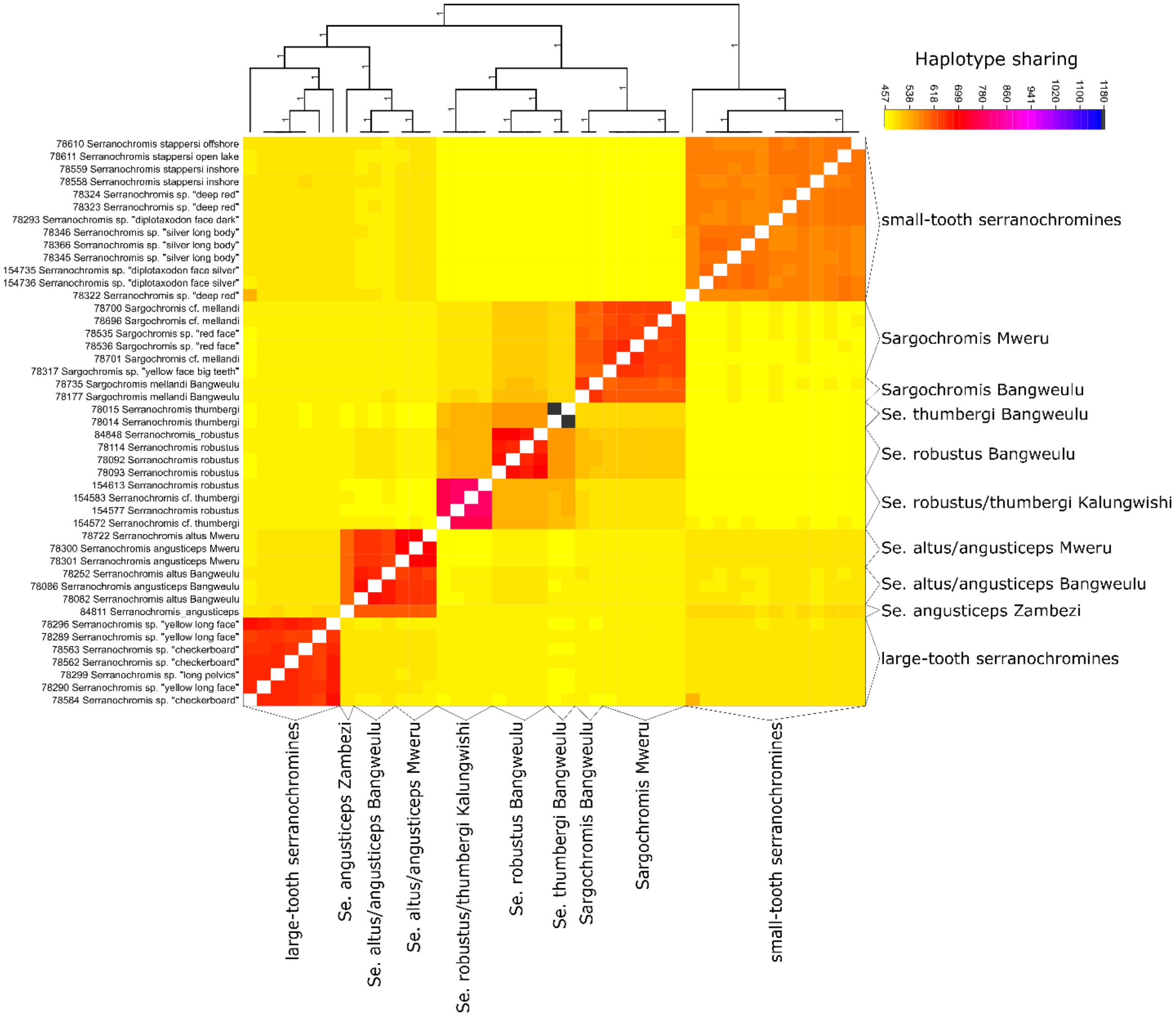

Supplementary Figure 9: fineRADstructure coancestry matrix of serranochromines reveals homogenous patterns of haplotype sharing within each radiation and except for one putative case of backcrossing, shows no evidence for recent gene flow. We used fineRADpainter to infer the closest relative (donor) for each haplotype (two per individual per RADlocus), and then ran fineRADstructure to infer the coancestry matrix. Donor individuals are shown in columns and recipient individuals are shown in rows. Ancient hybridization events inferred with D statistics and other methods are supported by fineRADstructure with weak haplotype sharing e.g. between small-tooth serranochromines and both large-tooth serranochromines and *Se. altus/angusticeps* and between *Se. robustus* Bangweulu and *Sargochromis* of both lakes. The fineRADstructure output files and the R script used to produce this figure is provided on Zenodo doi:10.5281/zenodo.3435419.

Supplementary Table 1: Mitochondrial chronogram calibration sets and age estimates. Calibration sets used for inference of the dated mitochondrial tree and estimates obtained for the age of the four Lake Mweru radiations. All dates are given in millions of years. For ages of deepest mitochondrial splits in each radiation, median and 95% highest posterior distributions are given. Note, the *Sargochromis* radiation in Lake Mweru shows incomplete haplotype sorting with *Sargochromis* individuals from Lake Bangweulu indicating that the radiation is younger than the node age. The full trees are provided on doi:10.5281/zenodo.3435419.

	i) lacustrine paleoenvironmental calibration	ii) fossil calibration scheme C10	iii) non-cichlid fossil calibration	iv) Gondwanan fragmentation calibration
References for calibration	Sturmbauer *et al.*[2] Koblmüller *et al.*[3]	Irisarri *et al.*[4]	Friedman *et al.*[5] Genner *et al.*[6]	Genner *et al.*[7] Genner *et al.*[6]
„modern haplo-chromines“ prior	Normal prior on crown age, mean 3.4, SD 0.5, 95% CI 2.58-4.22	Normal prior on crown age (excl. Tropheini), mean 4.05, SD 1.5, 95%CI 1.58-6.52	Normal prior on crown age (excl. Tropheini), mean 2.8, SD 0.5, 95% CI 1.98-3.62	Normal prior on crown age (excl. Tropheini), mean 8.5, SD 2, 95% CI 5.2-11.8
Lake Malawi radiation crown age prior	Uniform prior, 0.57-1 (refilling of Lake Malawi)	Normal prior, mean 1.1, SD 0.4, 95% CI 0.45-1.77	Normal prior, mean 1.2, SD 0.5, 95% CI 0.378-2.02	Normal prior, mean 4.06, SD 1, 95% CI 1.9-6.2
Pseudocrenilabrus	0.27 [0.10,0.46]	0.30 [0.05,0.64]	0.35 [0.12,0.62]	1.04 [0.39,1.85]
O. kalungwishiensis-complex	1.2 [0.64,1.74]	1.30 [0.33,2.33]	1.57 [0.82,2.5]	4.55 [2.16,7.28]
O. sp. ‘red-cheek’ / *O.* sp. ‘Kalungwishi’	0.36 [0.10,0.64]	0.41 [0.09,0.90]	0.47 [0.16,0.86]	1.37 [0.43,2.67]
small-tooth serranochromines	0.72 [0.38,1.13]	0.79 [0.11,1.44]	0.94 [0.43,1.56]	2.71 [1.33,4.65]
Sargochromis (incl. Bangweulu inds)	0.30 [0.14,0.47]	0.33 [0.04,0.62]	0.39 [0.17,0.65]	1.16 [0.43,1.89]
large-tooth serranochromines	<0.43 [0.20,0.69]	<0.47 [0.09,0.93]	<0.56 [0.21,0.92]	<1.63 [0.61,2.72]
LVRS	0.41 [0.20,0.66]	0.42 [0.09,0.92]	0.54 [0.25,0.92]	1.59 [0.63,2.72]

Supplementary Notes

Supplementary Note 1: Background on Lakes Mweru and Bangweulu

Lakes Mweru and Bangweulu are two of the ten African Great lakes that together straddle a region of major tectonic activity with shifting boundaries between Africa's three major watersheds Nile, Congo and Zambezi (Fig. 1). Whereas haplochromine cichlid species diversity in rivers is usually very modest with typically one to three species in any one river[8], some of the lakes in the region feature the highest density of fish species per area of all freshwater systems in the world, due to their large radiations of haplochromine cichlids[1,9]. Despite their large size, and the presence of several haplochromine lineages, Lakes Bangweulu and Mweru were not known to host radiations. Lake Mweru, in the upper Congo/Luapula drainage (5,120 km^2 surface, max 27 m deep)[10]. It is larger but less deep than Lake Edward (2,600 km2), which has a radiation of ~60 species[11]. Nearby Lake Bangweulu (Zambia) is only slightly smaller with a permanent open water surface of ca. 3,000-5,000 km^2 (extending to 15,000 km^2 of swamps in the wet season)[12-14] and a maximum depth of 10 m[13]. It lies further upstream in the Luapula drainage and has few fish species that are not also found in Lake Mweru[15].

The initial formation events of Lake Mweru and Lake Bangweulu are estimated to around 1 to 3 Mya[16,17]. Lake Bangweulu possibly originated when the Chambeshi had its drainage reversed and became landlocked in the Pliocene[17,18]. It used to be much larger and was draining southward into the Zambezi until about 1 Mya, when its outflow, the Chambeshi River, was captured by the Luapula and started flowing northwest towards Lake Mweru and the Congo[17]. Lake Mweru, in contrast, was always part of the upper Congo catchment. It likely formed in the late Pleistocene through rifting[16]. About a million years ago it suddenly received input from the Paleo-Bangweulu-Zambezi system at the time of the Luapula-Chambeshi capture event. Two waterfalls and a series of rapids make upstream fish movement through the Luapula from Lake Mweru to Lake Bangweulu very difficult but do not fully preclude downstream migration[15].

Supplementary Note 2: Taxonomic diversity

In Lake Bangweulu we report the occurrence of the endemic *Tylochromis bangwelensis*, the three widespread *Coptodon rendalli*, *Tilapia sparmanni* and *Oreochromis macrochir*, and six different species of haplochromine cichlids, five of which are widespread in the Zambezi and

have close relatives in Lake Mweru: *Serranochromis robustus, Se. thumbergi, Se. angusticeps, Se. altus,* and *Pseudocrenilabrus philander.* The sixth species, *Sargochromis mellandi*, of Lake Bangweulu is closely related only to *Sargochromis* of Lake Mweru and the Luapula river which connects the two lakes. All of these identifications were confirmed by matching the mitochondrial sequences to previously published sequences[18,19]. Lake Bangweulu did not reveal any non-Zambezian mitochondrial clades in its haplochromines, and its only Congo-derived cichlid was *Tylochromis bangwelensis*.

In contrast, in Lake Mweru and its two affluents, the Kalungwishi and the Luongo (a tributary of the Luapula just upstream of Lake Mweru), we encountered more than 40 different cichlid species only some of which we could readily identify as described species (Fig. 1 and Supplementary Fig. 2). The latter included six non-haplochromines: The endemic *Tylochromis mylodon*, the upper Congo *Tylochromis regani*, the endemic *Oreochromis mweruensis* and the more widely spread *Tilapia ruweti*, *Tilapia sparmanni* and *Coptodon rendalli*. All others were haplochromines. The previously known haplochromines encompass *Serranochromis robustus*, *Se. thumbergi*, *Se. altus* and *Se. angusticeps*, all widely distributed in the Zambezi River system and beyond in southern Africa. Further, we found one Mweru endemic in the genus *Serranochromis*, *S. stappersi,* and three Mweru endemics in two distinct mitochondrial clades of the Congolese genus *Orthochromis: O. polyacanthus* in one, and *O. kalungwishiensis* and *O. luongoensis* in the other clade, where we found the former only in the lake and the latter two only in rivers (Fig. 1, Supplementary Fig. 2). All other putative species represented an unexpected array of diverse phenotypes, varying in size, morphology and coloration (Supplementary Fig. 2). They fell into five major groups defined by a few key morphological features. (1) Multiple species of *Orthochromis*, defined by very elongate but cylindrical bodies and absence of specific anal fin markings. (2) A large diversity of species resembling the genus *Pseudocrenilabrus*, either by virtue of having a bright red spot on the trailing edge of the anal fin of males, or lacking this spot entirely but being otherwise of somewhat *Pseudocrenilabrus*-like appearance (the species in the “firetail” complex). This group included two species that resembled the taxonomic description of *Thoracochromis moeruensis* (a Panafrican taxonomic genus known to be highly polyphyletic[20]) and several related but distinct forms. This group also included several small demersal species including the recently described *Pseudocrenilabrus pyrrhocaudalis*[21]. (3) The genus *Sargochromis*, defined by many distinct ocelli (‘egg spots’) on

the anal fin of males, deep bodies and generalized morphology of the oral jaws. (4) The genus *Serranochromis*, defined by many distinct ocelli on the anal fin of males, elongate bodies, and a distinctly predatory head morphology with long lower jaw and large canine teeth (we refer to them as '*Serranochromis* large-tooth'). (5) A group of *Serranochromis*-like fish with predatory facies but with very small teeth (we refer to them as '*Serranochromis* small-tooth') and variable body shapes.

Supplementary Note 3: Species diversity in the Lake Mweru radiations

Pseudocrenilabrus radiation

The *Pseudocrenilabrus* radiation (clade VII of Joyce et al.[18]) is composed of very small (3 cm total length) to small species, none exceeding 10 cm in total length (Supplementary Figs 2, 5). In contrast to the typically conserved ecology of *Pseudocrenilabrus* that are confined to wetlands elsewhere in Africa[22,23], in Lake Mweru they occupy all sampled habitats in the main lake from littoral vegetation to open sandy beaches, exposed rocky boulder shores, and offshore demersal deep water zones. We identified individuals to 15 putative species, including one which phenotypically resembles the widely distributed *P*. *philander* but was genomically distinct from al *P. philander* populations of elsewhere. Samples of all 15 putative species are included in our RAD dataset. ANOVA tests reveal a significant effect of species on the first five axes of the genomic PCA ($p<0.01$) and on the first three axes of the morphological PCA (p: $<2.2\times10^{-16}$, 0.0004, 4.6×10^{-9}). Hence, differentiation in this radiation is highly dimensional, more so then in most of the others (see below). Interestingly, the species cluster by macrohabitat (littoral, offshore and rocky-shore) in the genomic PCA and partially also in the morphological PCA (Supplementary Fig. 4d). We thus performed additional PCAs for each macrohabitat separately: The offshore group with six morphologically highly derived demersal/pelagic species (Supplementary Fig. 4g), the rocky shore group with four putative species assigned to the nominal genus *Thoracochromis* (Supplementary Fig. 4e), and the littoral group with five putative species of sand and weed dwellers (Supplementary Fig. 4f). In the morphological PCA, the factor 'species' explains significant fractions of the variance on the first three axes in the offshore group (p: 1.2x10-9, 0.003, 0.009), on the first two axes in the rocky shore group (p: 0.018, 0.012), and

on the first axis in the littoral group (p: 0.0006). Similarly, in the genomic PCA, the first three axes in the offshore group show a significant effect of species (p: 0.016, 0.00015, 0.0033), the first axis in the rocky shore group (p: 0.0004), and the first two axes in the littoral group (p: 4.4x10-9, 0.03). Particularly in the offshore group, male nuptial coloration is bright and diversified and clearly distinguishes the putative species.

Orthochromis **radiations**

The *Orthochromis* assemblage was composed of three deeply divergent mitochondrial lineages that predate Lake Mweru by far (Supplementary Fig. 3). They are associated with *O. polyacanthus*, *O. sp*. “red cheek” and the riverine *O. kalungwishiensis* complex respectively. The latter complex includes *O. kalungwishiensis* and *O. luongoensis* and the very recently described species *O. mporokoso* and *O. katumbii,* all from the two major affluents of Lake Mweru, the Kalungwishi and the Luongo River[24]. In our study, the kalungwishiensis-complex is represented by *O. kalungwishiensis* and *O. katumbii*. We found four distinct genomic *Orthochromis* lineages and they do not match the three mitochondrial lineages (Supplementary Fig. 3, Supplementary Data 1). All three mitochondrially defined lineages must have exchanged genes with one another and one lineage must have arisen by hybridization with *Pseudocrenilabrus* (“New Kalungwishi” clade). It is hence not completely clear how to delineate the local radiation(s) in this group. Nonetheless, five of the seven riverine species and the two lacustrine species share typical *Orthochromis* traits (Supplementary Fig. 2): they are small haplochromines (usually <80 mm total length, Supplementary Fig. 5) with an elongate body, a reduced swim bladder, a steeply decurved head and short to very short oral jaws with many densely spaced teeth in the outer row, suggesting highly adapted *Aufwuchs* and algal scrapers. The remaining two species of the Kalungwishi River are morphologically very different and much more like *Pseudocrenilabrus* (Supplementary Fig. 2). In fact, one of them (“New Kalungwishi II”) is so close to *Pseudocrenilabrus* in appearance that we thought it was a giant *Pseudocrenilabrus* until the genetic data revealed its real relationships. Both “New Kalungwishi” clade species combine a *Pseudocrenilabrus*-like phenotype with mitochondrial haplotypes closely related to the *O. kalungwishiensis* complex and a unique position in our genomic tree where they reside on a unique branch between the *O. kalungwishiensis* complex and *Pseudocrenilabrus* (Supplementary Fig. 3). ANOVA on the morphological PCA (Supplementary Fig. 4h) of

Orthochromis reveals a significant effect of species for the first two axes (p: PC1: 3.9×10^{-9}, PC2: 0.016). ANOVA tests show that species explain a very significant proportion of the variation on the first four genomic PC axes (p: PC1: 2.2×10^{-7}, PC2: 7.1×10^{-7}, PC3: 4.5×10^{-10}, PC4: 4.8×10^{-9}).

Sargochromis radiation

The *Sargochromis* radiation (mitochondrial clade I of Joyce et al.[18] is composed of medium-sized (adult sizes between 10 and 25 cm standard length), deep-bodied fishes with trophic adaptations ranging from rather generalized insectivorous to specialized snail crusher morphology (Supplementary Fig. 2). All individuals were found exclusively in littoral habitats, mostly near submerged or emergent vegetation, in lagoons and along the shores of the main lake. In the field we recognized five different putative species, most of which were subsequently confirmed as distinct based on analyses of linear morphometric distances. Additionally, linear morphometric distances revealed significant shape differences between individuals of what we thought was just one type by external morphology but that either had small or large oral teeth (Supplementary Fig. 4a). We generated RAD-sequencing data for four of these morphologically defined species. ANOVA tests were significant for the first two PC axes of the morphological PCA (p-values: PC1: 0.04, PC2: 2.3×10^{-6}) and for the second axis in the genomic PCA (p: 0.0008). The species (*S. sp.* “big miller”) that was genetically most distinct was also phenotypically most distinct.

Serranochromis ‘small-tooth’ radiation

The *Serranochromis* ‘small-tooth’ radiation is composed of medium-sized to large predators (Supplementary Fig. 5) with diverse morphologies and ecologies which we assigned to six species based on morphology and coloration (Supplementary Figs 3, 4b). Some are littoral or benthic ambush hunters, others are offshore more pelagic predators, and one resembles morphologically the paedophages (egg predators) of Lake Malawi and Lake Victoria. For both the genomic and the morphological PCA, ANOVA tests revealed a significant effect of species on the first axis (p: genomic PC1: 2.8×10^{-5} and morphological PC1: 1.2×10^{-7}).

Serranochromis ‘large-tooth’ radiation

The *Serranochromis* ‘large-tooth’ radiation (mitochondrial clade III of Joyce et al.[18]) is composed of medium-sized to large (>25 cm total length) fish predators (Supplementary Figs 2, 5). They were found inshore near submerged and emergent vegetation, at rocky shores and offshore in shallow areas over soft bottom. We classified them into four putative species. The first two axes of the morphological PCA showed significant effects of species (p: PC1: 0.00012, PC2: 0.0061, Supplementary Fig. 4c). ANOVA tests did not reveal a significant influence of species on the PC axes in our genomic data, but this is likely due to low power given the low number of partial genomes (7 individuals distributed over 3 species).

Supplementary Note 4: Comparison of Lake Mweru haplochromines to those of Lake Victoria

Lake Mweru’s large diversity in ecologically relevant shape variation resembles that observed in the classical radiations of Lakes Victoria and Malawi. Quantitative morphological analysis suggests that each of the four new radiations contains between four and 15 phenotypically distinct taxa (Supplementary Fig. 4), many exhibiting novel phenotypes not reported from anywhere else in the large distribution range of the founding lineages outside Lake Mweru, especially in *Pseudocrenilabrus* and *Orthochromis*. At the same time, some of the Lake Mweru species resemble taxa of unrelated lineages in other African radiations. For instance, the rock dwelling *Pseudocrenilabrus* sp. ‘pundamilia-like’ resembles the Lake Victoria rock cichlid genus *Pundamilia*[25], the demersal *Pseudocrenilabrus pyrrhocaudalis*-complex resembles Lake Victoria detritivores of the genus *Enterochromis*, and the rock scraper *Orthochromis sp.* “red-cheek” resembles Lake Tanganyika algae browsers of the Tropheini tribe in its dentition traits and subterminal mouth opening. This is consistent with the parallel evolution of convergent sets of ‘ecomorphs’ in similar environments that had been suggested for other cichlid radiations[26,27] and other organisms[28-32]. In contrast to their large morphological disparity, species within all radiations are closely related and mitochondrial lineage sorting is absent, consistent with recent and rapid radiations[33].

The age of the Lake Mweru radiation is likely similar to that average of the Lake Victoria Region Superflock (Supplementary Fig. 6). D-loop sequence divergence among individuals within the

Lake Mweru radiations is 1.06% (mean pairwise genetic distances calculated as uncorrected p-distances excluding samples outside of the majority clade: Se. small-tooth: p=1.74%, Se. large-tooth: p=1.04%, *Sargochromis*: 0.64%, *Pseudocrenilabrus*: 0.83%) which is similar to the mean distance between Lake Victoria and Lake Edward/Kivu/Albert/Saka haplochromines. The maximum pairwise p-distances in each Mweru radiation are 3.6% for *Pseudocrenilabrus*, 3.8% for *Se.* small-tooth, 2.1% for *Sargochromis*, 2.4% for *Se*. large-tooth, whereas the maximum divergence between Lake Victoria cichlids and other LVRS members is 4.1%.

The similar pairwise p-distances and age calibration to date the onset of each radiation suggest that the radiations occurred largely simultaneously in the last millennium. To the best of our knowledge, this is the first finding of multiple adaptive radiations of cichlids starting at the same time in the same lake under sympatric conditions. Except for a small radiation of tilapiines (3-4 spp) besides the large haplochromine radiation (> 500 spp) in Lake Malawi, all other comparably recent cichlid radiations in Lakes Victoria, Edward, Malawi represent but one radiation, albeit the radiation ancestor probably being a hybrid population between several colonizing species[4,34]. The only other case of multiple lineages radiating in the same lake is the polyphyletic cichlid assemblage of Lake Tanganyika, but it seems that the main radiations have taken place consecutively over many millions of years rather than simultaneously in the past million years[35-37]. Lake Mweru thus provides unique opportunities to study interactions of multiple co-occurring radiations.

Supplementary Note 5: Evidence for hybrid origins of the adaptive radiations in Lake Mweru

"Orthochromine" radiations

Both, in the genomic and the mitochondrial tree, the *Pseudocrenilabrus* radiation of Lake Mweru forms a well-supported monophyletic clade nested within a paraphyletic *P. philander* clade that includes deeply divergent *philander*-like allopatric taxa from different localities (Fig. 3, Supplementary Fig. 3). However, while all except one samples of the radiation are mitochondrially sister to riverine *P. philander* from the Cunene River/Western Zambezi, they are all sister to *P. philander* of Lakes Bangweulu and Chilwa in the nuclear tree. One individual is also mitochondrially sister to *P. philander* of Lake Bangweulu (Supplementary Fig. 3, Supplementary Data 1). This may suggest that the *Pseudocrenilabrus* radiation is derived from a Mweru lineage related to the Cunene/Western Zambezi *Pseudocrenilabrus* (representing the Unknown Congolese mitolineage in Fig. 1 and Supplementary Fig. 3) with additional ancestry contributions from the Bangweulu *Pseudocrenilabrus* after the Luapula-Chambeshi River capture event. An admixed ancestry between these two lineages is corroborated by D statistics, whereby the *Pseudocrenilabrus* radiation in Lake Mweru shows excess allele sharing with *Pseudocrenilabrus* both from the Cunene and Western Zambezi Rivers and from Lake Bangweulu (Fig. 3, Supplementary Data 7).

The admixture graph method MixMapper was used to infer ancestry proportions of the *Pseudocrenilabrus* radiation (Supplementary Data 9). MixMapper infers the ancestry of a focal taxon by first reconstructing a phylogenetic tree of all given ancestor surrogates and then fitting the position of the focal taxon as a mixture of two of the ancestor surrogates. MixMapper suggests that the *Pseudocrenilabrus* radiation from Lake Mweru is derived from 54-61% *P. philander* Cunene/Western Zambezi (ancestor surrogate for the Congolese mitolineage) and 39-46% *P. philander* from Lake Bangweulu.

Our tests of hybridization also reveal additional excess allele sharing between the *Pseudocrenilabrus* radiation and "New Kalungwishi", a Kalungwishi River species that resemble *Pseudocrenilabrus* phenotypically but is mitochondrially *Orthochromis*-like. However, this might reflect gene flow from *Pseudocrenilabrus* lineage into the "New Kalungwishi" clade (see below). MixMapper can only infer two ancestral lineages and would thus not detect contributions from a third ancestor such as *Orthochromis*. Therefore, an additional MixMapper run was performed

with only one *P. philander* Cunene/Western Zambezi as potential *Pseudocrenilabrus* ancestor. Here, MixMapper infers a very small contribution of "New Kalungwishi" to the *Pseudocrenilabrus* radiation of Lake Mweru (Supplementary Data 9).

The most basal group within the Mweru *Pseudocrenilabrus* radiation (*P*. sp. "philander"), which phenotypically resembles *P. philander*, shows no excess allele sharing with any other taxon if compared to the *Pseudocrenilabrus* radiation (Supplementary Data 7). This suggests that hybridization was ancestral and is not ongoing. All evidence taken together implies that the *Pseudocrenilabrus* radiation evolved after admixture between the lineage that lived in Lake Mweru before the Bangweulu river capture and the lineage that arrived from Bangweulu.

The nominal genus *Orthochromis* is widely distributed in the Congo basin and is polyphyletic (Fig. 3, Supplementary Fig. 3). The *Orthochromis* taxa of the Mweru catchment alone are also polyphyletic in the mitochondrial tree, falling in two clades spanning the entire depth of the African haplochromine cichlid tree. One of them (*O. polyacanthus* and *O. stormsi*) is sister to serranochromines, *Orthochromis* of the Malagarasi and the Lake Victoria, Malawi and Tanganyika haplochromines. The other one contains all other *Orthochromis* taxa of the Mweru catchment and the panafrican genus *Pseudocrenilabrus*. Several instances of strong discordance between the mitochondrial genealogy, phenotype and nuclear genome are apparent among the *Orthochromis* of the Lake Mweru drainage system (Supplementary Fig. 3). In the nuclear tree, *O. polyacanthus* is the direct sister to all other Mweru *Orthochromis* to the exclusion of the serranochromines and all Malagarazi/Tanganyika/Malawi/Victoria clades which are mitochondrially sister to *O. polyacanthus*. This is best explained by ancient hybridization in the Mweru region between the ancestor of *O. polyacanthus* and an early representative of the endemic Mweru *Orthochromis* lineage. Indeed, D statistics support excess allele sharing between *O. polyacanthus* with each taxon of the genomic clade including *O. kalungwishiensis*-complex, *O*. sp. "Kalungwishi" and *O*. sp. "red-cheek" if compared to either *Pseudocrenilabrus* taxon, or "New Lufubu" or the "New Kalungwishi" clade (Fig. 3, Supplementary Data 8). Compared to *Pseudocrenilabrus*, also both "New Kalungwishi" species show excess allele sharing with *O. polyacanthus* (Fig. 3, Supplementary Data 8). Even though the directionality and exact source of gene flow cannot be inferred well with this dataset, the most likely scenario is hybridization between the common ancestor of the *O. kalungwishiensis* complex or a close relative and *O. polyacanthus*.

The deepest mitochondrial split among the second group, the Mweru-endemic "orthochromines" including only *Orthochromis* and *Pseudocrenilabrus,* separates the group of *O. sp.* "red cheek" of the rocky shores of Lake Mweru and *O*. sp. "Kalungwishi" of the lower reaches of the Kalungwishi river from all others, followed by a lineage that is phenotypically very close to *Pseudocrenilabrus* ("New Lufubu") and lastly, a clade including two species that resemble *Pseudocrenilabrus* one of which looks like a giant *Pseudocrenilabrus* ("New Kalungwishi II") and the *O. kalungwishiensis*-complex (Supplementary Fig. 3). This latter clade is mitochondrially sister to the genus *Pseudocrenilabrus.* All these relationships are completely different in the RAD tree (Fig. 3, Supplementary Fig. 3). In the RAD tree, the members of the most ancient mitochondrial clade (*O*. sp. "red-cheek" and *O*. sp. "Kalungwishi") are very closely related to the *O. kalungwishiensis*-complex from the upstream reaches of the rivers and these are not closely related to *Pseudocrenilabrus* (Fig. 3, Supplementary Fig. 3). Instead the two lineages that resemble *Pseudocrenilabrus* in phenotype, the "New Kalungwishi" clade and "New Lufubu", are genomically close to *Pseudocrenilabrus.*

Within the "New Kalungwishi" lineage, we identified two species that are distinct from each other both in morphology and genomics (Supplementary Fig. 4h). These new species resemble *Pseudocrenilabrus* phenotypically much more than they resemble other *Orthochromis.* At least one of them looks essentially identical to a giant *P. philander*. Males have a bright red egg dummy in the trailing edge of the anal fin (Supplementary Fig. 2) a trait otherwise unique in and diagnostic for *Pseudocrenilabrus*. They are found in extensive flooded still-water habitats in the Kalungwishi and Luongo river basins. D statistics reveal that both "New Kalungwishi" taxa show slight excess allele sharing with *Pseudocrenilabrus* from Lake Mweru (compared to other *Pseudocrenilabrus* from Lake Bangweulu but not if compared to other *Pseudocrenilabrus*) and strong excess allele sharing with "New Lufubu" and most *Orthochromis* taxa (compared to all *Pseudocrenilabrus*, Fig. 2, Supplementary Data 8). This is consistent with their mitochondrial affinity with other *Orthochromis* and their phenotypic resemblance with *Pseudocrenilabrus*. Massive hybridization with *Pseudocrenilabrus* may thus have facilitated the remarkable radiation of *Orthochromis* into *Pseudocrenilabrus*-like niches and phenotypes in the lentic river habitats (Supplementary Fig. 4c). MixMapper suggests that both "New Kalungwishi" species are derived from admixed ancestry of 73-80% "New Lufubu" and 20-27% of *Pseudocrenilabrus* (Supplementary Data 9), whereas "New Lufubu" is modeled to be of hybrid origin between

Orthochromis and the “New Kalungwishi” clade (Supplementary Data 9). Given that the “New Kalungwishi” clade shows mitochondrial similarity with the O. kalungwishiensis-complex, both taxa seem to have some *Orthochromis* and some *Pseudocrenilabrus* ancestry contributions. “New Kalungwishi” and “New Lufubu” may both share the same admixed ancestry between *Orthochromis* and *Pseudocrenilabrus* but not form a sister group due to additional ancestry contributions of *Pseudocrenilabrus* into the “New Kalungwishi” clade and/or additional ancestry contributions of *Orthochromis* into “New Lufubu”.

Serranochromine radiations

Comparison of the mitochondrial genealogy and the nuclear tree based on RAD sequencing data reveals cytonuclear discordance at the root of all serranochromine radiations (Fig. 3, Supplementary Fig. 3). Mitochondrially, the ‘large-tooth’ radiation is nested within the *S. altus/angusticeps* clade forming a sister group to the *Sargochromis* clade and the ‘small-tooth’ radiation forms a clade that is deeply divergent from these and sister to *S. robustus* and *S. thumbergi*. In the nuclear tree, in contrast, the two *Serranochromis* radiations are much more closely related. Here the ‘small-tooth’ radiation is even closer to the *S. altus/angusticeps* clade than the ‘large-tooth’ radiation is (despite the latter being mitochondrially nested within *altus/angusticeps*). *S. robustus/thumbergi* is an outgroup to both radiations in the genomic data, and forms the sister group to the *Sargochromis* clade.

The mitochondrial phylogeny also reveals some mismatch between our taxonomic assignment and the mitochondrial haplotype (Supplementary Fig. 3, dashed green lines in Fig. 3). One individual placed among the ‘large-tooth’ samples was phenotypically assigned to the ‘small-tooth’ group. One individual that we identified as belonging to the ‘large-tooth’ group has mitochondrial haplotypes nested in the *Se.* altus/angusticeps group. One ‘large-tooth’ and two *Sargochromis* individuals are mitochondrially part of the ‘small-tooth’ group. A phylogenetic analysis with 1331 amplified fragment length polymorphisms (AFLP) obtained for these mitochondrially mismatched individuals plus three other individuals of each of the serranochromine clades confirms that all individuals with mitochondrial-phenotype mismatch are members of their phenotypically matching clade in their nuclear genome. Hence, these individuals neither represent misidentifications nor convergent phenotypic evolution but they point to past hybridization among the three serranochromine radiations.

ADMIXTURE revealed no evidence for ongoing hybridization except for one ‘small-tooth’ radiation member (*Se.* sp. “diplotaxodon-face”) which showed a small proportion of admixture from the ‘large-tooth’ radiation cluster and was located basally in the nuclear tree. However, this individual showed a high proportion of missing data (49%) which could bias cluster assignments. The lowest cross-validation error is observed at K=6 clusters, which separates all radiations (Supplementary Fig. 7). With 5 clusters, the ‘large-tooth’ serranochromine samples are modeled as clustering with *Sargochromis* including a small ancestry proportion of the ‘small-tooth’ radiation.

Tests of past hybridization (D statistics, or ABBA-BABA tests) reveal excess allele sharing between the ‘small-tooth’ radiation and *Se. altus/angusticeps* of Lake Mweru (compared to *Se. altus/angusticeps* of Lake Bangweulu: mean z=5.4, compared to the ‘large-tooth’ radiation: z=6.2, Supplementary Data 2, Fig. 3). In addition, the ‘large-tooth’ radiation shows excess allele sharing with the ‘small-tooth’ radiation in respect to the *Se. altus/angusticeps* clade (z=8.1). Both radiations show weak excess allele sharing with *Se. robustus/thumbergi* of the Kalungwishi River, one of the two major inflowing rivers of Lake Mweru, in comparison to *Se. robustus* and *Se. thumbergi* of Lake Bangweulu (z: small-tooth: 3.7; large-tooth: 2.9, Fig. 3, Supplementary Data 2). Highly consistent D statistics independent of the choice of individuals from the ‘large-tooth’ and the ‘small-tooth’ radiations (Supplementary Data 4) suggests that excess allele sharing patterns are due to ancestral hybridization prior to the onset of diversification, rather than ongoing or recent gene flow.

MixMapper infers both *Serranochromis* radiations to be a mixture of something related to *Se. angusticeps* of the Zambezi River (~95%) and *Se. robustus* of the Kalungwishi River (~5%, Supplementary Data 6) if the Mweru radiations are not provided as potential ancestor surrogates. The *Se. robustus* Kalungwishi may represent the ancestor surrogate for the Upper Congolese mitochondrial lineage found in the ‘small-tooth’ radiation. If the Mweru radiations are included as potential ancestor surrogates, the ‘small-tooth’ radiation is inferred to be a mixture of 82-94% ‘large-tooth’ radiation and some *Se. angusticeps* from the Zambezi River (Supplementary Data 6). Similarly, the ‘large-tooth’ radiation is inferred to be mostly of ‘small-tooth’ radiation ancestry (64-95%). The second ancestor is less clear, ranging from something related to the riverine *Serranochromis* to something related to the entire group of *Sargochromis* and riverine *Serranochromis* or the group of all *Se. robustus* and *Se. thumbergi*.

Next, we carried out tree-way mixture models with MixMapper, where one *Serranochromis* radiation is inferred to be a mixture of *Se. angusticeps* and *Se. robustus* as detailed above and the other radiation is modeled as a mixture between the former admixed radiation and a second parental lineage (Supplementary Data 6). In these three-way mixture models, the 'small-tooth' radiation was inferred to be of 81-92% 'large-tooth' radiation ancestry and some *Se. angusticeps* of the Zambezi River ancestry. The 'large-tooth' radiation is inferred to be of 81-92% 'small-tooth' radiation ancestry and some *Se. macrocephalus* of the Zambezi River. The residuals of three-way mixtures were smaller than in the two-way mixtures for both radiations indicating a better fit of the three-way mixtures, but the 95% confidence interval overlapped 0 indicating insufficient power to determine the best model.

In conclusion, consistent with patterns of excess allele sharing, MixMapper suggests that each of the two Lake Mweru *Serranochromis* radiations is derived from a mix of *Se. angusticeps/altus, Se. robustus* and the other *Serranochromis* radiation. *Se. robustus* and the other radiation may represent the Upper Congo mitochondrial lineage found in 'small-tooth' serranochromines which is mitochondrially closest to the *Se. robustus/thumbergi* clade. This is also consistent with the rare occurrence of mitochondrially mismatched individuals and cytonuclear discordance of the two radiations, whereby in the mitochondrial tree the two radiations are sister to the distantly related *Se. robustus* and *Se. angusticeps* clades respectively, whereas in the nuclear tree they form a group of closely related clades together with *Se. altus/angusticeps*.

In both the nuclear and in the mitochondrial tree, the *Sargochromis* radiation of Lake Mweru is closest to the *Sargochromis* species of Lake Bangweulu (Supplementary Fig. 3). However, tests of hybridization revealed excess allele sharing between the Mweru *Sargochromis* radiation and both serranochromine radiations of Lake Mweru (z=3.4 for 'small-tooth' and 3.1 for the 'large-tooth' radiation, Fig. 3, Supplementary Data 3). The signal of excess allele sharing with 'large-tooth' serranochromines is weaker than with 'small-tooth' serranochromines and disappears if the *Sargochromis* radiation is tested against *Se. robustus* of Lake Malawi. This may suggest that the direction of gene flow is from 'small-tooth' serranochromines into the *Sargochromis* radiation and that 'large-tooth' serranochromines show some excess allele sharing due to their close relationship to the 'small-tooth' serranochromines. Consistent with this hypothesis, all individuals of the 'small-tooth' radiation show highly consistent signals of excess allele sharing (Supplementary Data 4). However, if each *Sargochromis* individual is tested separately, two

samples do not show significant excess allele sharing with ‘small-tooth’ serranochromines. Interestingly, excess allele sharing with ‘small-tooth’ serranochromines is weakest for the individual of *Sa.* sp. “yellow face big teeth” (Supplementary Data 4) which is mitochondrially basal in the *Sargochromis* radiation (Supplementary Data 1). F4 tests of ‘small-tooth’ vs ‘large-tooth’ serranochromines and Sargochromis Mweru vs Bangweulu are not significant even though BABA patterns are a bit more frequent than ABBA patterns.

However, mitochondrial haplotype sharing supports admixture between ‘small-tooth’ serranochromines and *Sargochromis* of Lake Mweru. Two *Sargochromis* individuals have mitochondrial haplotypes that are closely related to each other and are nested among the ‘small-tooth’ serranochromines (Fig. 3, Supplementary Data 1). In the AFLP tree, they clearly fall into the *Sargochromis* Mweru clade. More samples will be needed to test if the entire species of Sa. sp. “yellow face big teeth” shows less evidence for ‘small-tooth’ serranochromine allele sharing of if it is just this sample.

Samples with phenotypic and mitochondrial clade affiliation are genomically assigned to their phenotypic group

As we started working on this collection of new cichlids, we came across some individuals with conspicuous mismatches between their mitochondrial haplotype clade affiliation and their phenotypic clade affiliation. Before the advent of Next Generation Sequencing techniques, we generated multilocus AFLP genotypes for these individuals to test if these were cases of taxonomic misidentification, phenotypic convergent evolution or hybridization. For comparison we also included individuals for which phenotype and haplotype clade affiliations were in agreement. The distantly related species *Thoracochromis demeusii* (from the lower Congo River) and *Metriaclima estherae* (endemic to Lake Malawi) were used as outgroups. The lab protocol and phylogenetic methods followed Joyce et al.[18]. The DNA of these samples was used up in the process precluding them from inclusion in the RAD dataset. In the AFLP tree, all samples fell into the respective groups that matched their phenotype, revealing that all of these samples showed a cyto-nuclear mismatch. There was no case of taxonomic misidentification and no evidence for phenotypic convergence.

The divergence time of the colonizing lineages of Lake Mweru provide opportunity for hybridization

The Lake Mweru radiations are unique among African cichlid radiations in several ways. First, the genus *Pseudocrenilabrus*, which impressively diversified in Lake Mweru, also occurs in wetlands around all other African Great lakes, but has not invaded the open lake anywhere else, nor has it speciated anywhere else[9,22]. Second, Lake Mweru also accommodates several other radiations of completely unrelated lineages. Other lakes typically have only one radiating lineage. The multiple colonists of Lake Mweru represent ecologically and phenotypically very different types. It seems plausible that the initial occupation of divergent ecological niches in a newly formed lake allows for the coexistence of most of the ancestral types, providing multiple starting points in niche space for subsequent episodes of adaptive radiation. Competition for resources between the main ecological guilds would then have constrained the directions and set the boundaries to phenotypic diversification within each radiation.

In principle, the same pre-radiation conditions existed in Lakes Victoria, Malawi, Edward and Tanganyika. Each of these lakes was colonized by several, deeply divergent haplochromine lineages besides the one that radiated: *Pseudocrenilabrus* colonized all lakes, *Serranochromis* colonized Lakes Malawi and Tanganyika, *Astatoreochromis* colonized Lakes Victoria, Edward and Tanganyika. Yet none of these colonists radiated. One difference is that in these lakes, in contrast to Lake Mweru, all of these colonists were phylogenetically too isolated to hybridize with each other, except for multiple *Astatotilapia/'Thoracochromis'* species that did merge into a single hybrid population from which all radiations in the Lake Victoria region emerged[34]. The divergence times between all the other colonists[7] fall well outside the experimentally determined window of opportunity for hybridization[38]. The same explains why serranochromines and "orthochromines" did not hybridize in Lake Mweru despite much sympatry, and would potentially explain why *Pseudocrenilabrus* radiated in Lake Mweru but not in Lake Bangweulu.

Supplementary References

1 Wagner, C. E., Harmon, L. J. & Seehausen, O. Cichlid species-area relationships are shaped by adaptive radiations that scale with area. *Ecol Lett* **17**, 583-592 (2014).

2 Sturmbauer, C., Baric, S., Salzburger, W., Rüber, L. & Verheyen, E. Lake level fluctuations synchronize genetic divergences of cichlid fishes in African lakes. *Mol Biol Evol* **18**, 144-154 (2001).

3 Koblmüller, S. *et al.* Age and spread of the haplochromine cichlid fishes in Africa. *Mol Phylogenet Evol* **49**, 153-169 (2008).

4 Irisarri, I. *et al.* Phylogenomics uncovers early hybridization and adaptive loci shaping the radiation of Lake Tanganyika cichlid fishes. *Nat Commun* **9**, 3159 (2018).

5 Friedman, M. *et al.* Molecular and fossil evidence place the origin of cichlid fishes long after Gondwanan rifting. *Proc R Soc B* **280**, 20131733 (2013).

6 Genner, M. J., Ngatunga, B. P., Mzighani, S., Smith, A. & Turner, G. F. Geographical ancestry of Lake Malawi's cichlid fish diversity. *Biol Lett* **11**, 20150232 (2015).

7 Genner, M. J. *et al.* Age of cichlids: New dates for ancient lake fish radiations. *Mol Biol Evol* **24**, 1269-1282 (2007).

8 Seehausen, O. Process and pattern in cichlid radiations–inferences for understanding unusually high rates of evolutionary diversification. *New Phytol* **207**, 304-312 (2015).

9 Seehausen, O. African cichlid fish: a model system in adaptive radiation research. *Proc R Soc B* **273**, 1987-1998 (2006).

10 Bos, A. R., Kapasa, C. K. & van Zwieten, P. A. M. Update on the bathymetry of Lake Mweru (Zambia), with notes on water level fluctuations. *Afr J Aqua Sci* **31**, 145-150 (2006).

11 Bezault, E., Mwaiko, S. & Seehausen, O. Population genomic tests of models of adaptive radiation in Lake Victoria Region cichlid fish. *Evolution* **65**, 3381-3397 (2011).

12 Herdendorf, C. E. Large lakes of the world. *J Great Lakes Res* **8**, 379-412 (1982).

13 Shiklomanov, I. A. & Rodda, J. C. *World water resources at the beginning of the twenty-first century*. (Cambridge University Press, 2004).

14 Bos, A. R. & Ticheler, H. A limnological update of the Bangweulu fishery, Zambia. *DoF/BF/1996/Report no.26, Dept. of Fisheries Zambia* (1996).

15 Van Steenberge, M., Vreven, E. & Snoeks, J. The fishes of the Upper Luapula area (Congo basin): a fauna of mixed origin. *Ichthyol Explor Freshw* **24**, 289-384 (2014).

16 Cotterill, F. & De Wit, M. Geoecodynamics and the Kalahari epeirogeny: linking its genomic record, tree of life and palimpsest into a unified narrative of landscape evolution. *S Afr J Geol* **114**, 489-514 (2011).

17 Stankiewicz, J. & de Wit, M. J. A proposed drainage evolution model for Central Africa—Did the Congo flow east? *J Afr Earth Sci* **44**, 75-84 (2006).

18 Joyce, D. A. *et al.* An extant cichlid fish radiation emerged in an extinct Pleistocene lake. *Nature* **435**, 90-95 (2005).

19 Katongo, C., Koblmüller, S., Duftner, N., Mumba, L. & Sturmbauer, C. Evolutionary history and biogeographic affinities of the serranochromine cichlids in Zambian rivers. *Mol Phylogenet Evol* **45**, 326-338 (2007).

20 Schwarzer, J. *et al.* Repeated trans-watershed hybridization among haplochromine cichlids (Cichlidae) was triggered by Neogene landscape evolution. *Proc Biol Sci* **279**, 4389-4398 (2012).

21 Katongo, C., Seehausen, O. & Snoeks, J. A new species of Pseudocrenilabrus (Perciformes: Cichlidae) from Lake Mweru in the Upper Congo River System. *Zootaxa* **4237**, 181-190 (2017).
22 Katongo, C., Koblmüller, S., Duftner, N., Makasa, L. & Sturmbauer, C. Phylogeography and speciation in the Pseudocrenilabrus philander species complex in Zambian Rivers. *Hydrobiologia* **542**, 221-233 (2005).
23 Greenwood, P. H. Towards a phyletic classification of the 'genus' *Haplochromis* (Pisces, Cichlidae) and related taxa. Part 1. *Bull Br Mus Nat Hist Zool* **35**, 265-322 (1979).
24 Schedel, F. D. B., Vreven, E., Manda, B. K., Abwe, E. & Schliewen, U. K. Description of five new rheophilic Orthochromis species (Teleostei: Cichlidae) from the Upper Congo drainage in Zambia and the Democratic Republic of the Congo. *Zootaxa* **4461**, 301-349 (2018).
25 Seehausen, O. *Lake Victoria Rock Cichlids. Taxonomy, Ecology and Distribution*. (1996).
26 Fryer, G. & Iles, T. D. *The cichlid fishes of the great lakes of Africa: their biology and evolution*. (Oliver and Boyd, 1972).
27 Greenwood, P. H. The cichlid fishes of Lake Victoria, East Africa: the biology and evolution of a species flock. *Bulletin of the British Museum of Natural History (Zoology Series)* **Suppl. 6**, 1-134 (1975).
28 Schluter, D. Ecological speciation in postglacial fishes. *Philosophical Transactions of the Royal Society of London Series B-Biological Sciences* **351**, 807-814, doi:10.1098/rstb.1996.0075 (1996).
29 Losos, J. B., Jackman, T. R., Larson, A., de Queiroz, K. & Rodriguez-Schettino, L. Contingency and determinism in replicated adaptive radiations of island lizards. *Science* **279**, 2115-2118 (1998).
30 Gillespie, R. Community assembly through adaptive radiation in Hawaiian spiders. *Science* **303**, 356-359 (2004).
31 Madsen, O. *et al.* Parallel adaptive radiations in two major clades of placental mammals. *Nature* **409**, 610 (2001).
32 Bossuyt, F. & Milinkovitch, M. C. Convergent adaptive radiations in Madagascan and Asian ranid frogs reveal covariation between larval and adult traits. *Proceedings of the National Academy of Sciences* **97**, 6585-6590 (2000).
33 Schluter, D. *The ecology of adaptive radiation*. (Oxford University Press Inc., 2000).
34 Meier, J. I. *et al.* Ancient hybridization fuels rapid cichlid fish adaptive radiations. *Nat Comm* **8**, 14363 (2017).
35 Salzburger, W., Mack, T., Verheyen, E. & Meyer, A. Out of Tanganyika: Genesis, explosive speciation, key-innovations and phylogeography of the haplochromine cichlid fishes. *BMC Evol Biol* **5** (2005).
36 Salzburger, W., Meyer, A., Baric, S., Verheyen, E. & Sturmbauer, C. Phylogeny of the Lake Tanganyika Cichlid species flock and its relationship to the Central and East African Haplochromine Cichlid fish faunas. *Syst Biol* **51**, 113-135 (2002).
37 Takahashi, K., Terai, Y., Nishida, M. & Okada, N. Phylogenetic relationships and ancient incomplete lineage sorting among cichlid fishes in Lake Tanganyika as revealed by analysis of the insertion of retrotransposons. *Mol Biol Evol* **18** (2001).
38 Stelkens, R. B., Young, K. A. & Seehausen, O. The accumulation of reproductive incompatibilities in African cichlid fish. *Evolution* **64**, 617-632 (2010).
