# Supplementary material for: The coincidence of ecological opportunity with hybridization explains rapid adaptive radiation in Lake Mweru cichlid fishes
Source: Nat Commun. 2019 Dec 3;10:5391. doi: 10.1038/s41467-019-13278-z (PMC6890737; doi:10.1038/s41467-019-13278-z)
Supplement: Supplementary file 3 — Reporting Summary [file 41467_2019_13278_MOESM3_ESM.pdf]

nature research

Corresponding author(s): Ole Seehausen

Last updated by author(s): Sep 26, 2019

Reporting Summary

Nature Research wishes to improve the reproducibility of the work that we publish. This form provides structure for consistency and transparency in reporting. For further information on Nature Research policies, see Authors & Referees and the Editorial Policy Checklist.

Statistics

For all statistical analyses, confirm that the following items are present in the figure legend, table legend, main text, or Methods section.

n/a	Confirmed	
☐	☒	The exact sample size (*n*) for each experimental group/condition, given as a discrete number and unit of measurement
☒	☐	A statement on whether measurements were taken from distinct samples or whether the same sample was measured repeatedly
☐	☒	The statistical test(s) used AND whether they are one- or two-sided<br>*Only common tests should be described solely by name; describe more complex techniques in the Methods section.*
☐	☒	A description of all covariates tested
☐	☒	A description of any assumptions or corrections, such as tests of normality and adjustment for multiple comparisons
☐	☒	A full description of the statistical parameters including central tendency (e.g. means) or other basic estimates (e.g. regression coefficient) AND variation (e.g. standard deviation) or associated estimates of uncertainty (e.g. confidence intervals)
☐	☒	For null hypothesis testing, the test statistic (e.g. *F*, *t*, *r*) with confidence intervals, effect sizes, degrees of freedom and *P* value noted<br>*Give P values as exact values whenever suitable.*
☐	☒	For Bayesian analysis, information on the choice of priors and Markov chain Monte Carlo settings
☐	☒	For hierarchical and complex designs, identification of the appropriate level for tests and full reporting of outcomes
☒	☐	Estimates of effect sizes (e.g. Cohen's *d*, Pearson's *r*), indicating how they were calculated

Our web collection on statistics for biologists contains articles on many of the points above.

Software and code

Policy information about availability of computer code

Data collection	n/a
Data analysis	All software used is mentioned in the manuscript and own scripts are provided on GitHub or on Zenodo: doi:10.5281/zenodo.3435419.

For manuscripts utilizing custom algorithms or software that are central to the research but not yet described in published literature, software must be made available to editors/reviewers. We strongly encourage code deposition in a community repository (e.g. GitHub). See the Nature Research guidelines for submitting code & software for further information.

Data

Policy information about availability of data

All manuscripts must include a data availability statement. This statement should provide the following information, where applicable:

- Accession codes, unique identifiers, or web links for publicly available datasets
- A list of figures that have associated raw data
- A description of any restrictions on data availability

Mitochondrial sequences newly generated for this study have been uploaded to GenBank with accession numbers MN167933 - MN168155. Raw reads of RAD data are available at the NCBI Sequence Read Archive under Bioproject PRJNA553794. The morphology data, the D statistics used for plotting, the genomic data files used for generating PCA plots, the fineRADstructure output files, the BEAST trees with all calibration sets, the RAD variant calls, and the mitochondrial and RAD RAxML trees are provided on Zenodo doi:10.5281/zenodo.3435419. This is also where the R scripts using these files to produce Figs 2-4, Supplementary Figs 4,5,8,9, and Supplementary Data 1 are located. The fully labelled trees underlying Supplementary Figure 2 are given as Supplementary Data 1. All D statistics, f4 test results and MixMapper results are given as Supplementary Data files 2-10. Information on the samples used for mitochondrial sequencing, morphological measurements, and RAD sequencing are provided as Supplementary Data 11, 12, and 13, respectively.

Field-specific reporting

Please select the one below that is the best fit for your research. If you are not sure, read the appropriate sections before making your selection.

☐ Life sciences ☐ Behavioural & social sciences ☒ Ecological, evolutionary & environmental sciences

For a reference copy of the document with all sections, see nature.com/documents/nr-reporting-summary-flat.pdf

Ecological, evolutionary & environmental sciences study design

All studies must disclose on these points even when the disclosure is negative.

Study description	We sampled cichlid fishes in two African lakes (Mweru and Bangweulu) to test if there were indeed no adaptive radiations as the literature had suggested. Once we detected that Lake Mweru harbours multiple adaptive cichlid radiations, we produced morphological measurements to study their ecology and adaptations, and genomic data to infer their evolutionary relationships, age, and history of hybridization and to compare the Mweru cichlid radiations with the cichlids in Lake Bangweulu and some surrounding rivers where no adaptive radiations occurred.
Research sample	Haplochromine cichlid fishes caught in Lakes Mweru and Bangweulu, and some surrounding rivers.
Sampling strategy	We collected cichlids from as many different habitats and sites as possible to assure a good representation of the diversity of cichlid fish in the lakes and to confirm the presence or absence of adaptive radiations in each lake. We collected and preserved a total of 404 specimens from Lake Bangweulu, more than 1,000 specimens from Lake Mweru and about 50 specimens from the rivers. For the genomic data analysis only a subset of the fish specimens was used to have more or less even sampling of the different groups.
Data collection	Cichlids were collected with beach seines, gill nets, by angling or bought on the local fish market. Some samples were obtained from collaborators. where possible, live fish were photographed, a fin clip was taken for DNA extraction and the body was fixed in formalin and then transferred to 75% ethanol for the best preservation of morphological traits.
Timing and spatial scale	Between September and October 2005, we sampled eleven locations along the southern and eastern coasts of Lake Mweru. Three of these sites were sampled again in 2017. Sampling sites included shallow, sandy beaches in the south, a rocky outcrop on Kilwa Island in the south-west, steeply sloping, wave exposed beaches and rocky boulder shores in the northeast, and offshore open waters in the south, south-west and northeast. We also sampled two sites in the large lagoon network directly south of Lake Mweru. In 2005 we sampled the lower reaches of the Kalungwishi River. In 2017 we sampled 5 sites in the middle and upper reaches of the Kalungwishi River. In Lake Bangweulu, we sampled five locations, including white sandy beaches, large water lily beds, and extensive reed swamps.
Data exclusions	Samples with too much missing data (>50%) in the genomic dataset were excluded from all analyses except phylogenetic tree inference.
Reproducibility	Multiple samples of each species and radiation were used to ensure that e.g. signatures of hybridization are not due to sampling a single hybrid individual but indeed are a characteristic of the entire species or adaptive radiation. All data is uploaded to Short Read Archive (RAD sequences) or GenBank (mitochondrial sequences). R scripts and their input files to produce the figures are provided on Zenodo: doi:10.5281/zenodo.3435419.
Randomization	Samples were assigned to groups based on morphological traits and colouration. Only adult fish were considered.
Blinding	No blinding required.

Did the study involve field work? ☒ Yes ☐ No

Field work, collection and transport

Field conditions	Most sampling was carried out in 2005 or 2017 under various field conditions.
Location	Lake Mweru: 8.9368° S, 28.7664° E<br>Lake Bangweulu: 11.2044° S, 29.7426° E
Access and import/export	Preserved samples were exported as scientific material for research purposes from Zambia to Switzerland using courier services provided by DHL or FedEx after obtaining written permission from the Department of Fisheries of Zambia.
Disturbance	Beach seining was carried out by teams of professional fishermen as part of their routine fishing. Gillnets were set by ourselves and were left in the water only for a short amount of time to obtain all fish in good condition and to cause minimum disturbance. Other methods do not cause disturbance.

Reporting for specific materials, systems and methods

We require information from authors about some types of materials, experimental systems and methods used in many studies. Here, indicate whether each material, system or method listed is relevant to your study. If you are not sure if a list item applies to your research, read the appropriate section before selecting a response.

Materials & experimental systems

n/a	Involved in the study
☒	☐ Antibodies
☒	☐ Eukaryotic cell lines
☒	☐ Palaeontology
☐	☒ Animals and other organisms
☒	☐ Human research participants
☒	☐ Clinical data

Methods

n/a	Involved in the study
☒	☐ ChIP-seq
☒	☐ Flow cytometry
☒	☐ MRI-based neuroimaging

Animals and other organisms

Policy information about studies involving animals; ARRIVE guidelines recommended for reporting animal research

Laboratory animals	The study did not involve laboratory animals.
Wild animals	Cichlids were collected with beach seines, gill nets, by fishing or bought on the local fish market. Some samples were obtained from collaborators. If possible, life fish were photographed, then euthanized with an overdose of MS222. A fin clip was taken for DNA extraction and the body was stored in formalin and then transferred to 75% ethanol for best preservation of morphological traits.
Field-collected samples	The cichlids were humanely killed immediately after capture.
Ethics oversight	No ethical guidance required as the fish were immediately killed humanely and not used for experiments.

Note that full information on the approval of the study protocol must also be provided in the manuscript.
