# Supplementary material for: The coincidence of ecological opportunity with hybridization explains rapid adaptive radiation in Lake Mweru cichlid fishes
Source: Nat Commun. 2019 Dec 3;10:5391. doi: 10.1038/s41467-019-13278-z (PMC6890737; doi:10.1038/s41467-019-13278-z)
Supplement: Supplementary file 4 — Description of Additional Supplementary Files [file 41467_2019_13278_MOESM4_ESM.pdf]

Description of Additional Supplementary Files

File Name: Supplementary Data 1
Description: Comparison of nuclear (RAD) and mitochondrial (D-loop) phylogenetic trees with individual sample labels. The symbols correspond to Supplementary Fig. 2 which shows a simplified version of the trees.

File Name: Supplementary Data 2
Description: D statistics testing for hybridization involving the two Serranochromis radiations

File Name: Supplementary Data 3
Description: D statistics testing each individual of the serranochromine radiations separately show high consistency among individuals

File Name: Supplementary Data 4
Description: D statistics testing hybridization involving the Sargochromis radiation of Lake Mweru

File Name: Supplementary Data 5
Description: f4 tests for hybridization among serranochromines

File Name: Supplementary Data 6
Description: MixMapper results for serranochromine radiations

File Name: Supplementary Data 7
Description: D statistics testing for hybridization involving the Pseudocrenilabrus radiation of Lake Mweru

File Name: Supplementary Data 8
Description: D statistics testing for excess allele sharing between Orthochromis and Pseudocrenilabrus

File Name: Supplementary Data 9
Description: MixMapper to infer ancestry of Orthochromis and Pseudocrenilabrus groups

File Name: Supplementary Data 10
Description: Lake Bangweulu taxa do not show evidence for excess allele sharing with any other taxa compared to their Lake Mweru drainage sister taxa (except for Se. robustus)

File Name: Supplementary Data 11
Description: Samples used for mitochondrial DNA sequences

File Name: Supplementary Data 12
Description: Morphological measurements

File Name: Supplementary Data 13
Description: Samples used for RAD sequencing
