# Supplementary figures and images for: The coincidence of ecological opportunity with hybridization explains rapid adaptive radiation in Lake Mweru cichlid fishes
Source: Nat Commun. 2019 Dec 3;10:5391. doi: 10.1038/s41467-019-13278-z (PMC6890737; doi:10.1038/s41467-019-13278-z)

## RAD

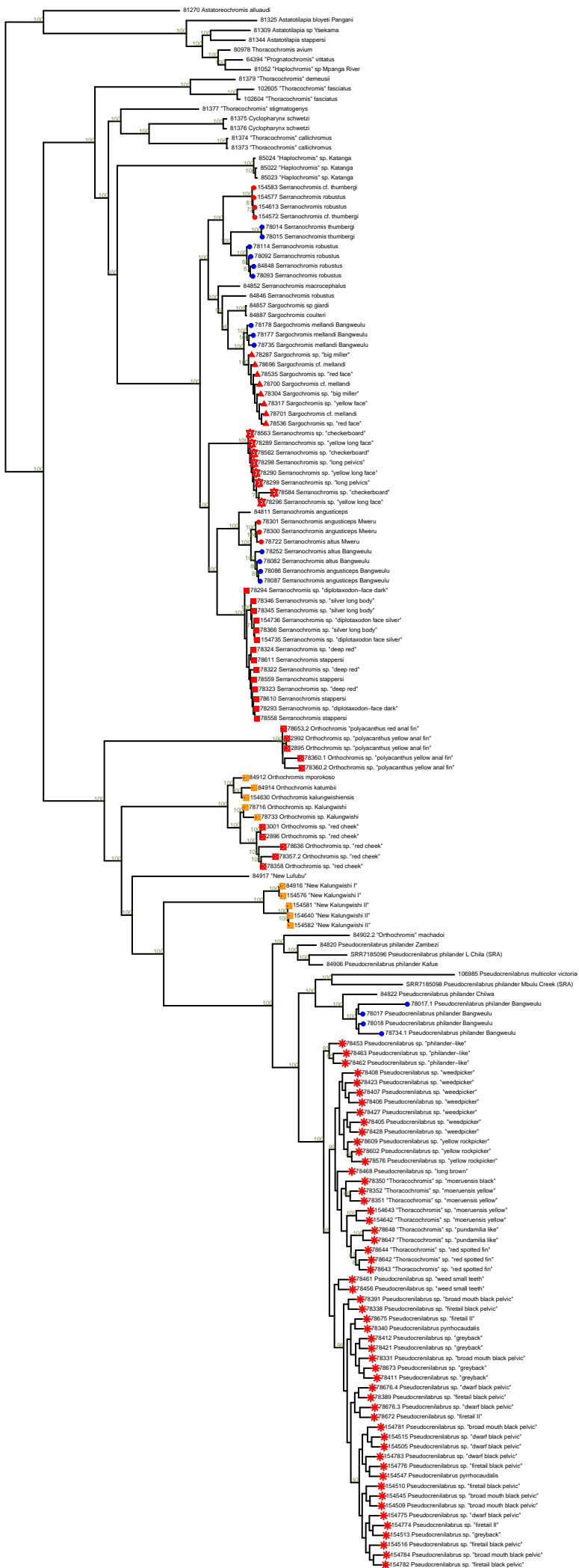

## mtDNA

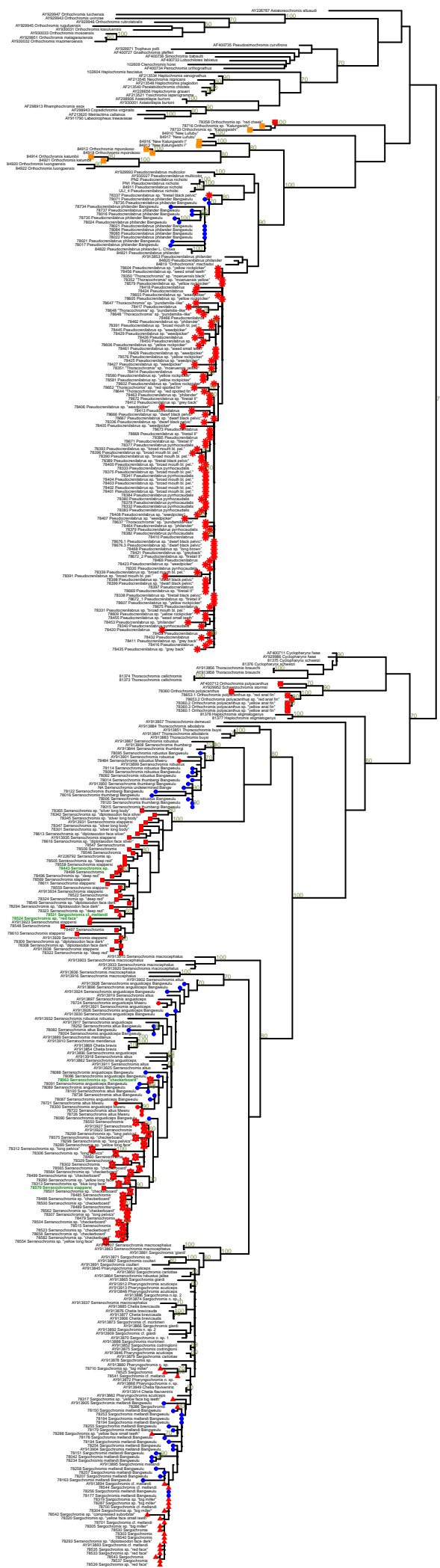

Supplement: Supplementary file 5 — Supplementary Data 1 [file 41467_2019_13278_MOESM5_ESM.pdf]
